# Epidemiology, clinical manifestations, and laboratory findings of 1,590 human brucellosis cases in Ningxia, China

**DOI:** 10.3389/fmicb.2023.1259479

**Published:** 2023-11-27

**Authors:** Bofei Liu, Guangtian Liu, Xueping Ma, Fang Wang, Ruiqing Zhang, Pan Zhou, Zhiguo Liu, Zhenjun Li, Xuefeng Jiang

**Affiliations:** ^1^Department of Communicable Control and Prevention, The Fourth People's Hospital of Ningxia Hui Autonomous Region, Yinchuan, China; ^2^Department of Virology Laboratory, Ningxia Hui Autonomous Region Center for Disease Control and Prevention, Yinchuan, China; ^3^National Institute for Communicable Disease Control and Prevention, Chinese Center for Disease Control and Prevention, Beijing, China

**Keywords:** human brucellosis, zoonosis, clinical symptoms, laboratory findings, orchitis, spondylitis, treatment outcome

## Abstract

**Introduction:**

Epidemiological and clinical analyses of brucellosis are vital for public health leaders to reinforce disease surveillance and case management strategies.

**Methods:**

In this study, we aimed to analyse the epidemiology and clinical features of 1,590 cases of human brucellosis.

**Results:**

Approximately 72.08% (1,146) of the patients were male and 27.92% (444) were female. At least 88.18% (1,402/1,590) of the patients had a history of contact with sheep/goats and cattle, which was identified as the main risk factor for infection. The most common age group affected was 30–69 years, comprising 83.90% of all cases, with a median age of 47.3 years. Meanwhile, 75.03% (1,193/1,590) of the patients were farmers, followed by workers (10.50%, 167/1,590). The spectrum of clinical manifestations varied, and the major symptoms were fatigue (42.96%), joint pain (37.30%), and fever (23.33%). Arthritis was diagnosed in 989 patients, spondylitis was diagnosed in 469 patients, and external genital complications were found in at least 53.96% (858/1,590) of patients. In addition, approximately 41.25% (625/1,515) and 24.53% (390/1,590) of cases exhibited elevated CRP and D-dimer levels, respectively. Conversely, a significant decrease was observed in fibrinogen, total protein, and albumin levels, affecting 48.36% (769/1,590), 77.30% (1,226/1,586), and 91.80% (1,456/1,586) of the patients, respectively. These data demonstrate that brucellosis is a severe wasting disease that leads to an imbalance in nutritional metabolism and a decline in immunity. In total, 86.73% (1,379/1,590) of patients showed improvement with antibiotic therapy, while 13.27% (211/1,590) of patients experienced relapses or treatment failure.

**Conclusion:**

Brucellosis often presents with non-specific symptoms and laboratory findings, accompanied by multiple organ invasions, as well as being a vital challenge for diagnosis and treatment; thus, it is essential for a high degree of suspicion to be placed on brucellosis for a timely diagnosis and treatment. This study provides basic data and resources for developing tailored countermeasures to curb its further spread.

## Introduction

Human brucellosis, caused by *Brucella* spp., is a global public health concern (Pappas et al., 2006; Di Bonaventura et al., 2021). In 1884, the causative bacteria *Brucella* was first discovered in the spleen of a soldier who died from the infection (Galińska and Zagórski, 2013). These bacteria are small, aerobic, gram-negative rods belonging to the genus *Brucella* with an incubation period typically ranging from 5 to 60 days, mostly 1–2 months (Song et al., 2021). Small ruminants and bovines, which excrete the bacteria in milk and reproductive discharges, are major sources of infection for humans (Khan et al., 2021), and contact with contaminated animals and consumption of unpasteurised dairy products are the main routes for human infection (Bagheri Nejad et al., 2020).

Currently, the *Brucella* genus contains 12 species—six classical and six atypical (Occhialini et al., 2022); the recent study revealed that two distinct isolates from humans in French Guiana represent a new species of *Brucella*, which proposes the name *Brucella amazoniensis* sp. nov. (About et al., 2023). In humans, the disease is mainly caused by *Brucella melitensis*, the most pathogenic species, followed by *Brucella suis*, whereas *Brucella abortus* is considered the mildest type of brucellosis (Galińska and Zagórski, 2013; Rabah et al., 2022). Brucellosis significantly affects human health and the animal farming industry because *Brucella* strains give rise to severe clinical manifestations in humans, such as fever, sweating, and fatigue, as well as vast economic losses from animal farming due to problems such as abortion and a decline in milk productivity (Bagheri Nejad et al., 2020; Jamil et al., 2021). Although some developed countries have nearly eliminated this disease, most low-income countries, particularly in Africa, Latin America, and Asia— notably China—still face significant challenges regarding this zoonosis (Gwida et al., 2010; Zhu et al., 2020; Yawoz et al., 2021). Brucellosis has been reported in all 32 mainland provinces of China but is particularly prevalent in the northwest and northeast regions, where animal husbandry is the primary industry (Yang et al., 2023); for example, the reported incidence rate dramatically increases from 7.0/100,000 in 2005 to 23.5/100,000 in 2014 in Shanxi, showing an average annual increase of 14.5% (Chen et al., 2016).

Ningxia Hui Autonomous Region (Ningxia) is located in northwest China. Since 1995, human brucellosis has re-emerged in this region owing to changes in agricultural practices, population and animal movements, and healthcare access (Lai et al., 2017). By 2022, more than 1,590 patients with brucellosis had been admitted to hospitals for treatment. An analysis of the epidemiological and clinical traits of cases in regions experiencing a re-emergence of brucellosis is essential to ensure that healthcare providers and public health leaders are aware of the current trends of brucellosis and to reinforce disease surveillance and case management (Franco et al., 2007; Yagupsky et al., 2019; Matle et al., 2021). This study seeks to address a critical knowledge gap by conducting a comprehensive analysis of the epidemiological and clinical characteristics of human brucellosis in Ningxia, shedding light on previously unexplored aspects of the disease's resurgence in this region and providing valuable insights for improving prognosis, decreasing chronic disease, and reducing relapse.

## Methods

### Data source, diagnosis, processing, and analysis

Between January 2019 and December 2022, 1,590 patients with brucellosis, presenting with or without complications, were admitted to the Department of Infectious Diseases at the Fourth People's Hospital. Among these patients, data from 1,590 cases of human brucellosis were retrospectively collected and analyzed. Clinical data collected include demographic information (sex, age, and nationality) and epidemiological characteristics (contact types) (Table 1), clinical manifestations and complications, physical examination findings, laboratory and imaging findings, treatment history, and records of relapse. Patient data were obtained from electronic medical records, including records of relapse, via a telephone interview. Excel 2020 software was used to clean, process, and statistically analyse the data.

**Table 1:** Demographics, age group, occupation, and contact history of brucellosis cases.

**Key**	**Items**	**Epidemiology indexes**	**No**.	**Proportion (%)**
1	Staging	Acute	979	61.6%
		Chronic	611	38.4%
2	Sex	Male	1,146	72.08%
		Female	444	27.92%
3	Age	~9	12	0.75%
		~19	35	2.20%
		~29	157	9.87%
		~39	231	14.53%
		~49	380	23.90%
		~59	487	30.63%
		~69	236	14.84%
		~79	49	3.08%
		80–83	3	0.19%
4	Occupation	Farmers	1,193	75.03%
		Workers	167	10.50%
		Veterinarian	52	3.27%
		Pupil	32	2.01%
		Entity	29	1.82%
		Housework and unemployment	21	1.32%
		Unknown	18	1.13%
		Retired worker	14	0.88%
		Unemployed	12	0.75%
		Retiree	10	0.63%
		Rural laborer	9	0.57%
		Cadre staff	7	0.44%
		Other	7	0.44%
		Scattered children	6	0.38%
		Teacher	5	0.31%
		Herdsman	3	0.19%
		Civil servant	2	0.13%
		Policeman	1	0.06%
		Medical personnel	1	0.06%
		Nursery child	1	0.06%
5	Contact history	Contacted with animals	1,402	88.18%
		Uncontacted with animals	186	11.70%
		Unknown	2	0.13%

### Case definition and diagnosis metrics

The diagnosis of human brucellosis was based on serological tests, clinical manifestations, and epidemiological information. The Rose Bengal plate test (RBPT) and serum agglutination test (SAT) were utilized for brucellosis screening and diagnosis (Araj, 2010). A diagnosis of brucellosis was confirmed when the RBPT yielded positive results and the standard tube agglutination test showed a titer of ≥1:100, or when the duration of the disease course was more than 1 year with a titer of ≥1:50. Cultures of blood or other tissue samples are not routinely performed at this hospital due to biosafety concerns (Liu et al., 2022). According to previous reports, brucellosis cases were divided into acute (< 6 months) and chronic phases (>6 months) based on the duration of clinical symptoms (Jiang et al., 2019). Upon hospitalization, each brucellosis case underwent a comprehensive assessment, including detailed epidemiological contact history, physical examination, and biochemical analyses (blood cell count, routine biochemical parameters, and routine urinalysis). The laboratory values reported in the text and tables refer to the results of the patient's first examination. Additional imaging examinations such as ultrasonography, CT, MRI, echocardiography, and scrotal Doppler ultrasonography were conducted for patients with complications. Relapse was defined as the reappearance of symptoms or a 4-fold increase in SAT titres within 6 months of therapy (Buzgan et al., 2010).

## Results

### Demographics and geographic distribution of brucellosis cases

Among the 1,590 patients, there were more number of male (1,146, 72.08%) than female (444, 27.92%) individuals (Table 1). The age range of the patients ranged from 1 to 83 years, and most of the patients (83.90%) were between the ages of 30 and 69 years. Furthermore, the distribution of patients by age category was assessed as follows: 30–39, 14.53%; 40–49, 23.90%; 50–59, 30.63%; and 60–69, 14.84% (Table 1). The median age of the patients was 47.3 years. The majority of patients (75.03%, 1,193/1,590) were farmers, followed by workers (10.50%, 167/1,590), veterinarians (3.27%, 52/1,590), and pupils (2.01%, 32/1,590). Other occupations accounted for < 2.0% of the study population (Table 1). At least 88.18% (1,402/1,590) of the cases reported contact with sheep/goats and cattle, and 11.70% (186/1,590) denied having had any contact with domestic animals (sheep and cattle) (Table 1). The majority of cases (96.73%) were registered from Ningxia, whereas only 3.27% were recorded in other surrounding areas [Inner Mongolia (*n* = 26), Gansu (*n* = 13), Shaanxi (*n* = 11), Xinjiang (*n* = 1), and Henan (*n* = 1)]. Our analysis showed that most patients were living in rural areas, where animals and people living in close contact may favor the spread of brucellosis.

### Clinical symptoms and diagnostic indices in brucellosis cases

Based on the serological test results, RBPT was positive in all 1,590 patients with suspected brucellosis, and SAT was employed for further examination. All patients tested positive for SAT, and the anti-Brucella serum antibody titres ranged from 1:100 to 1:800. Only 8.18% (130/1,590) of the cases had no complications, and more than 91.82% (1,460/1,590) of the cases had complicated brucellosis. The clinical symptoms of human brucellosis can vary widely and include fatigue, sweating with fever, headache, backache, lumbosacral pain, right chest pain, shortness of breath, left leg pain with lameness, body ache, chronic joint pain, testicular swelling and pain with fever, intermittent fever with lower back and knee pain, and multiple wandering pains throughout the body. Among these symptoms, fever, joint pain, and fatigue were the most common and accounted for 23.33% (371/1,590), 37.30% (593/1,590), and 42.96% (683/1,590) of cases, respectively (Table 2). Approximately 59.57% (221/371) of the patients exhibited intermittent low-grade fever. Osteoarticular system involvement was a vital complication of brucellosis, including arthritis in 989 (62.20%) and spondylitis in 469 (29.49%) patients. Notably, 337 patients had both arthritis and spondylitis. In total, 53.96% (858/1,590) of the patients were diagnosed with external genital complications. At least 50.57% (804/1,590) of the cases showed involvement of the testis and epididymis, with 804 cases reporting testicular involvement and 328 cases presenting with clinical signs related to the epididymis. The most common clinical symptom associated with the testis was hydrocele (*n* = 780), followed by orchitis (*n* = 50), testicular cysts (*n* = 33), and bilateral hydrocele (*n* = 32) (Table 2). Epididymal cysts were the most common clinical symptom in epididymis-involved cases, accounting for 91.46% (300/328) of all cases (Table 2). A total of 274 patients showed clinical manifestations in both the epididymis and testis, with 246 of them presenting with hydroceles of the testis and epididymal cysts (Table 2). Additionally, various other complications were observed among the patients, including hypoproteinaemia in 579 patients, leukopenia in 219, mild anemia in 130, hypoproteinaemia complicated with hypokalaemia in 96, pericardial effusion in 50, cervical cysts in 39, and moderate anemia in 19 patients. In 67 cases, co-infections of brucellosis and tuberculosis were diagnosed (Table 2). Our data showed that brucellosis can cause a devastating multi-organ disease in humans with serious health complications.

**Table 2:** Clinical symptoms and complications profile of human brucellosis cases in this study.

**Classification**	**Items**	**No**.	**Proportion (%)**
Symptoms	Fever	371	23.33%
	Intermittent fever	221	13.90%
	Joint pain	593	37.30%
	Fatigue	683	42.96%
	Sweat	130	8.18%
Complications	Spondylitis	469	29.50%
	Brucella encephalitis	1	0.06%
	Brucellosis arthritis	989	62.20%
	Brucellosis arthritis with spondylitis	337	21.19%
	Hypoproteinaemia	579	36.42%
	Leukopenia	219	13.77%
	Mild anemia	130	8.18%
	Hypoproteinaemia with hypokalaemia	96	6.04%
	Tuberculosis	67	4.21%
	Pericardial effusion	50	3.14%
	Cervical cyst	39	2.45%
	Moderate anemia	19	1.19%
	Meningitis	13	0.82%
	External genital complications	858	53.96%
	Testicle involved	804	50.57%
	Orchitis	50	3.14%
	Testicular cyst	33	2.08%
	Hydrocele of testicle	780	49.06%
	Bilateral hydrocele	32	2.01%
	Epididymis involved	328	20.63%
	Cyst of epididymis	300	18.87%
	Cyst of epididymis with hydrocele of vaginalis	246	15.47%
	Epididymis + testicles	274	17.23%

### Laboratory findings in 1,590 human brucellosis cases

Table 3 presents the results of the laboratory tests. Approximately 41.25% (625/1,515) exhibited elevated CRP levels, with an average CRP level of 31.52 mg/L (tested range of value as 10.1–106.7) (Table 3). In the case of the D-dimer test, 24.53% (390/1,590) of patients had values higher than the normal reference value (ref. =1.0 μg/ml), with a median value of 2.3 μg/ml (Table 3). Additionally, fibrinogen (FIB) examination revealed a decline in 48.36% (769/1,590) of the cases, with a median value of 1.27 g/L (Table 3). Approximately 31.89% (507/1,590) of patients exhibited abnormal hemoglobin (HGB) levels, with 308 patients showing elevated levels and 199 patients showing decreased levels (Table 3). Furthermore, 77.30% (1,226/1,586) and 91.80% (1,456/1,586) of patients had decreased total protein (TP) and albumin (ALB), respectively (Table 3). The present data showed that brucellosis is a severe wasting disease that leads to an imbalance in nutritional metabolism and a decline in immunity.

**Table 3:** Laboratory findings of patients with brucellosis.

**Classification**	**Variable**	**Reference range** ^*^		**No. (%)**	**Median**
Inflammation test	CRP	0–10 mg/L		41.25% (625/1,515)	31.52
	ESR	Female	0–20 mm/h	74.39% (61/82)	48.4 mm/h
		Male	0–15 mm/h	69.05% (116/168)	40.8 mm/h
	PCT	0.01–0.5 ng/ml	>0.25	3.06% (10/327)	0.33
			>0.5	1.83% (6/327)	0.92
Coagulation function test	PT%	70%−150%	< 70%	56	60.90%
			>150%	4	/
	D-dimer	0–1 μg/ml		24.53% (390/1,590)	2.3
	FIB	2–4 g/L	< 2	48.36% (769/1,590)	1.27
			>4	10.31% (164/1,590)	6.6
Blood routine examination	WBC	3.5–9.5 × 10^9^	>9.5	5.28% (84/1,590)	10.9
	HGB	115–150 g/L	< 115	12.53% (199/1,588)	109
			>150	19.40% (308/1,588)	583
	PLT	125–350 × 10^9^ L	< 125	8.38% (133/1,588)	90.3
			>350	2.64% (42/1,588)	375.8
Liver function test	ALT	7–50 U/L	< 7	3.34% (53/1,587)	5.2
			>50	8.63% (137/1,587)	111.3
	AST	13–40 U/L	< 13	22.68% (360/1,587)	11.4
			>40	9.26% (147/1,587)	97.6
	TBIL	2–21 μmol/L	< 2	1.45% (23/1,587)	1.5
			>21	5.67% (90/1,587)	30.5
	DBIL	0–4 μmol/L		14.68% (233/1,587)	10.4
	IDBIL	0–17 μmol/L		6.74% (107/1,587)	21.7
	TP	65–85 g/L	< 65	77.30% (1,226/1,586)	59.25
	ALB	40–55 g/L	< 40	91.80% (1,456/1,586)	33.8
Renal function test	BUN	1.7–7.1 mmol/L	>7.1	3.72% (59/1,585)	9.2
	CREA	44–133 μmol/L	< 44	9.15% (145/1,584)	37.84
	UA	208.3–428.4 μmol/L	< 208	35.04% (555/1,584)	170.86

^*^Referred to normal reference range of diagnosis indices.

### Treatment situation and outcome

Among the 1,590 patients, 1,379 received one treatment, 159 received two treatments, 35 received three treatments, 11 received four treatments, four received five treatments, and two received six treatments. The most commonly used treatment regimes consisted of rifampicin, doxycycline, and levofloxacin, and ~86.73% (1,379/1,590) of patients exhibited significant improvement, while 13.27% (211/1,590) of patients experienced relapses or treatment failure during follow-up after the end of treatment.

Patients with tuberculosis who were co-infected with brucellosis were treated with rifampicin, isoniazid, pyrazinamide, and amikacin. Meningitis cases were managed with rifampicin, doxycycline, and cephalosporin. Some chronic patients were treated with Chinese medicine formulas, such as Manna Disinfection Dan. Additionally, various adjuvant therapies were employed, including drugs such as glycyrrhizin, gluconolactone tablets, bicyclol, and Shensong Yangxin capsules (Chinese medicine). Following treatment, routine blood tests, as well as liver and renal function tests were reviewed every 2–4 weeks. Anteroposterior and lateral lumbar radiographs, bilateral knee ultrasound, and color ultrasound of the scrotum, testis, and epididymis were reviewed after 2 months. The physiological and biochemical indexes of the majority of patients returned to normal to varying degrees. Our analysis showed that antibiotic therapy combined with other treatment regimens could rapidly improve the signs and symptoms of the infection.

## Discussion

In this study, we retrospectively analyzed the epidemiology, clinical characteristics, and laboratory findings of 1,590 cases of human brucellosis. Our analysis showed that male patients comprised the majority of the infected population, and at least 88.18% (1,402/1,590) of the cases had a history of contact with sheep (goats) and cattle. The predominance of men in the labor force can be attributed to the region's economic reliance on agriculture, particularly in Ningxia, a renowned hub for domestic farming. For instance, the “Yanchi Tan Sheep,” a domestic farming product, has earned national recognition as a registered brand. The livestock breeding industry consistently serves as a vital component of the local economy; however, activities such as processing, selling, and cooking of mutton can lead to infection in humans. In 2022, mutton production in Ningxia reached 124,800 tons, representing the highest recorded growth rate (8.8%) among all meat types compared to 2021. In Xinjiang, 77.1% of male brucellosis cases and 60.5% of the total cases reported close contact with cattle or sheep. These data revealed that direct or indirect domestic contact poses a significant risk for infection, including activities such as assisting with deliveries, shearing, and handling aborted fetuses and their secretions (Soi et al., 2010).

In this study, the most common age group affected was 30–69 years, accounting for 83.90% of all cases, with a median patient age of 47.3 years. In contrast, in southern Saudi Arabia, the highest prevalence was observed in the 21–40 years age group (40.5%), followed by the 41–60 years age group (27.7%) (Alkahtani et al., 2020). In Spain, male patitents under 45 years of age and of urban origin were found to be at a higher risk of brucellosis (Rodríguez-Alonso et al., 2021). The spectrum of clinical manifestations varies widely, with the major symptoms identified in this study being fatigue, joint pain, and fever. A previous study showed that fatigue was the most common symptom in the Xinjiang Uygur Autonomous Region, in contrast to fever, which was the most common clinical manifestation of brucellosis in Anhui Province (Shi et al., 2021). A systematic review of the clinical manifestations of human brucellosis found that fever, arthralgia, myalgia, and back pain were the dominant symptoms (Dean et al., 2012). These findings highlight the challenge of accurately diagnosing human brucellosis and the importance of combining contact history and laboratory results for diagnosis.

Our analysis also revealed a high proportion of osteoarticular complications. Osteoarticular involvement was the most common complication associated with brucellosis, affecting 10–85% of the infected patients across various provinces, with the sacroiliac (up to 80%) and spinal joints (up to 54%) being the most commonly affected sites (Esmaeilnejad-Ganji and Esmaeilnejad-Ganji, 2019). Therefore, a combination of radiography, computed tomography (CT), magnetic resonance (MR) imaging, and bone scintigraphy is necessary for diagnosis. Surgical treatment is also required for spondylitis and paravertebral or epidural abscesses (Jia et al., 2023).

Brucellosis often leads to genitourinary complications in the endemic regions (Savasci et al., 2014). In this study, the testis and epididymis were involved in at least 50.57% (804/1,590) of cases. A previous study reported that the incidence of epididymal orchitis in human brucellosis was 7.6% (69/912), whereas another study documented genitourinary complications in 27 male patients with brucellosis at a university hospital in Diyarbakir from 1998 to 2006 (Celen et al., 2010). Genitourinary brucellosis is common among infected humans in endemic areas and is considered the second most affected focal site, commonly manifesting as epididymal orchitis (Hamoda et al., 2023). Epididymo-orchitis has also been reported in childhood brucellosis (Oguz and Oztek-Celebi, 2018). Our findings highlight the importance of echographic examination for diagnosing granulomatous epididymal orchitis and avoiding unnecessary orchiectomy for benign disease (Salmeron et al., 1998). The high proportion of genitourinary complications of brucellosis in this study requires further investigation as they may be linked to areas where brucellosis re-emerges and doctors are relatively unfamiliar with the disease. Therefore, clinicians encountering epididymal orchitis in brucellosis-endemic areas should consider the possibility of brucellosis as a potential diagnosis.

The CRP assay is a valuable index for diagnosing and monitoring the treatment of patients with brucellosis (Hashemi et al., 2018; Xu et al., 2020). Our analysis revealed that ~41.25% (625/1,515) of patients had elevated CRP levels. A retrospective evaluation and review of the clinical manifestations and complications in 1,028 cases of brucellosis showed that the most common laboratory finding was a high C-reactive protein level (Buzgan et al., 2010). Another study showed that mean serum CRP levels in patients were significantly higher than those in the control group (Hashemi et al., 2018). Patients with complicated brucellosis tend to have higher CRP levels than those with uncomplicated brucellosis (Xu et al., 2020), and elevated CRP levels are significantly associated with brucellosis complications (Yetkin et al., 2005). The data from this study strongly support the conclusion that CRP levels are valuable diagnostic indicators for early brucellosis screening. Additionally, 24.53% (390/1,590) of patients exhibited high D-dimer levels. A study on COVID-19 co-infection in a patient with brucellosis reported a high D-dimer concentration (Shabani and Ghadimi, 2022). Another case report showed *B. melitensis* isolated from blood cultures and a D-dimer value of 1,056 mug/L (50–228 mug/L) (Turunc et al., 2008). A study on a brucellosis case with disseminated intravascular coagulation showed plasma fibrinogen levels of 20 mg/dl, and D-dimer levels of 8 μg/ml (Akbayram et al., 2011). These data indicate the need for further evaluation of the role of D-dimer in the diagnosis of brucellosis.

Brucellosis is a multi-organ invading disease that affects almost all organs, as well as physiological and biochemical indicators in the body at varying degrees, including immunity (inflammation), coagulation function, routine blood indicators, liver function, and renal function. Notably, we found a significant decrease in FIB, TP, and ALB levels. Similarly, in a case of disseminated intravascular coagulation caused by *B. melitensis*, FIB was too low to be detected in the serum of the patient, and 4 days after antibiotic treatment was administered, all hematological abnormalities were normalized (Turunc et al., 2008). Moreover, some patients exhibited elevated HGB and UA (uric acid) levels. These data indicate that brucellosis is a severe wasting disease that leads to an imbalance in nutritional metabolism and a decline in resistance. Timely treatment and adequate nutrition are necessary to improve patient outcomes. Both drugs and diseases have adverse effects on liver and renal function; therefore, while treating brucellosis, attention should be paid to the protection of liver and renal function. The recommended regimens for brucellosis treatment involve two or three antibiotic combinations, and the most common combination used in the current study was doxycycline plus rifampicin.

The findings highlight various shortcomings in current prevention and treatment strategies, and a major limitation of the study is insufficient data on whole-course follow-up after the end of treatment. It is therefore recommended that epidemiological follow-up of public health diseases such as brucellosis be made mandatory in order to better highlight the impact of the disease over time and use this information in order to apply the most adequate infection control policies. Second, the study's retrospective design may introduce bias and limitations in data collection and accuracy, and the study's findings are based on available medical records, and missing or incomplete data can affect the accuracy and comprehensiveness of the analysis. Finally, the study relies on documented clinical manifestations and laboratory findings, which may not capture all symptoms or complications experienced by patients; some individuals may not seek medical attention or may not be correctly diagnosed.

## Conclusion

The clinical and laboratory characteristics of human brucellosis have been described in order to assist clinicians in early diagnosis of and monitoring the disease. Brucellosis produces varying degrees of adverse effects on immunity, coagulation function, routine blood indicators, liver function, and renal function. Furthermore, CRP levels and D-dimer assays can be helpful indicators for diagnosing and monitoring human brucellosis. Osteoarticular and external genital involvement were the main complications associated with brucellosis. Our findings emphasize the importance of follow-up with brucellosis patients after the end of treatment to better evaluate the impact of the disease over time and to apply the most adequate infection control policies. Meanwhile, the implementation of strict intervention strategies and increased diagnostic capabilities for brucellosis are urgently needed.

## Data availability statement

The original contributions presented in the study are included in the article/supplementary material, further inquiries can be directed to the corresponding authors.

## Ethics statement

The studies involving humans were approved by the Ethics Committee of the Ningxia Fourth People's Hospital (No. NXFPH-0624). The studies were conducted in accordance with the local legislation and institutional requirements. Written informed consent for participation in this study was provided by the participants' legal guardians/next of kin.

## Author contributions

BL: Formal analysis, Writing—review & editing, Conceptualization. GL: Investigation, Writing—review & editing, Data curation, Methodology, Resources. XM: Data curation, Investigation, Methodology, Resources, Writing—review & editing. FW: Data curation, Formal analysis. RZ: Investigation, Methodology, Writing—review & editing. PZ: Writing—review & editing, Formal analysis, Project administration, Visualization. ZLiu: Investigation, Visualization, Writing—original draft, Writing—review & editing. ZLi: Methodology, Resources, Writing—review & editing. XJ: Conceptualization, Writing—review & editing.
